# Differentiating Pseudo Versus True Aortic Stenosis in Patients Without Contractile Reserve: A Diagnostic Dilemma

**DOI:** 10.7759/cureus.14086

**Published:** 2021-03-24

**Authors:** Khushal V Choudhary, Nikolaos Kakouros, Gerard P Aurigemma, Matthew W Parker, Timothy Fitzgibbons

**Affiliations:** 1 Department of Internal Medicine, Roger Williams Medical Center, Providence, USA; 2 Cardiovascular Division, Department of Medicine, University of Massachusetts Medical School, Massachusetts, USA

**Keywords:** low flow, low gradient, aortic stenosis, transcutaneous aortic valve replacement, dobutamine, ischemia

## Abstract

Low-flow, low-gradient (LF-LG) aortic stenosis with depressed left ventricular (LV) ejection fraction is a diagnostic challenge that is frequently encountered in the management of valvular heart disease. True-severe LF-LG aortic stenosis is amenable to valve replacement, whereas pseudo-severe aortic stenosis requires management of the underlying cardiomyopathy. This distinction is important as it serves as a critical branch point in guiding therapeutic decisions.

We present the case of a 71-year-old male with LF-LG aortic stenosis who had a reduced and biphasic augmentation of LV flow during dobutamine stress echocardiography (DSE). Further evaluation revealed a stenotic left subclavian artery proximal to the left internal mammary artery graft to the left anterior descending (LAD) artery. Bypass of the subclavian stenosis reversed the LAD territory ischemia and confirmed pseudo-severe aortic stenosis on repeat DSE.

Traditional DSE parameters are inconclusive in patients with LF-LG aortic stenosis with poor flow reserve. Calculation of the projected orifice area or measurement of aortic valve calcium via multidetector computed tomography (MDCT) may be required in this scenario. Most importantly, reversible causes of LV dysfunction identified during DSE for LF-LG aortic stenosis require a different treatment approach than that of true aortic stenosis.

## Introduction

Approximately 30% of patients with low-flow, low-gradient (MG [mean gradient] < 40 mmHg; EOA [effective orifice area] < 1.0 cm²; EF [ejection fraction] < 50%; AVAi [aortic valve area index] < 0.6 cm²/m²) aortic stenosis (AS) have pseudo-severe AS (PSAS) [1]. In patients with sufficient flow reserve, defined as a 20% increase in stroke volume, dobutamine stress echocardiography (DSE) can be helpful to differentiate true-severe AS (TSAS) from PSAS. However, in patients without flow reserve, differentiating PSAS from true AS remains challenging and may require innovative approaches. The main objective of our case report is to differentiate between true and pseudo AS and to demonstrate the utility of dobutamine at different dosing in the diagnosis of true versus pseudo AS.

## Case presentation

We present the case of a 71-year-old man with a past medical history of coronary artery disease, remote history of three-vessel coronary artery bypass graft surgery (LIMA-LAD [left internal mammary artery-left anterior descending artery], SVG-PDA [saphenous vein graft to the posterior descending artery, SVG-OM [SVG to the obtuse marginal branch of the circumflex]), ischemic cardiomyopathy, hypertension, hyperlipidemia, and paroxysmal atrial fibrillation. He presented to the hospital following four weeks of progressive weakness, shortness of breath, and a syncopal episode. His vitals revealed a blood pressure of 110/64 mg Hg, pulse of 70 bpm, respiratory rate of 12 breaths per minute, and SaO_2_ 100% on 4 liters supplemental oxygen by nasal cannula. On physical examination, he was cachectic and chronically ill in appearance, with peripheral cyanosis, pallor, and elevated jugular venous pressure. There was a mid to late peaking systolic ejection murmur at the base that was 3/6 in intensity and radiated to the carotid arteries. His carotid pulse was weak but not delayed.

**Figure 1:**
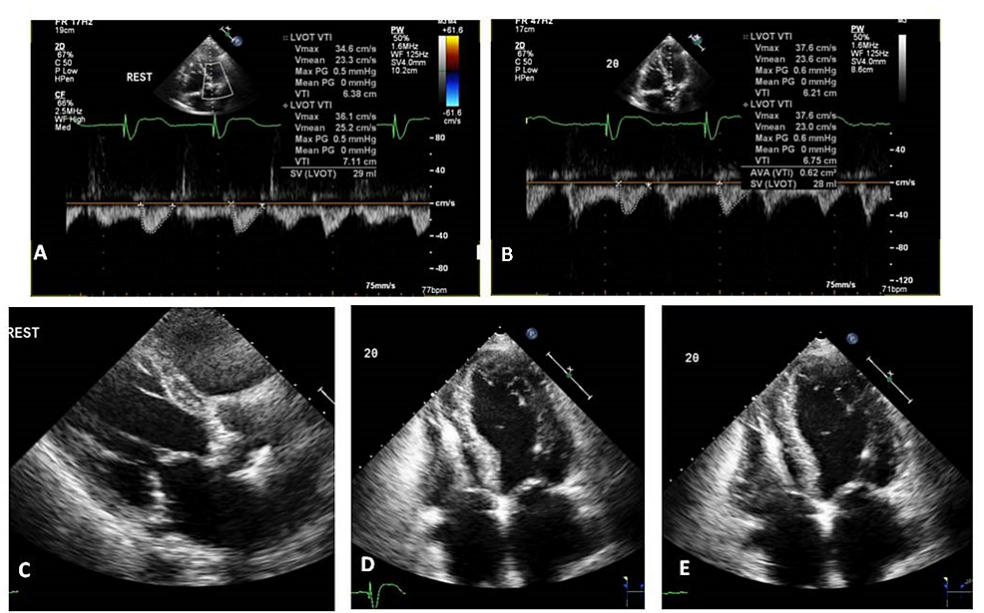
Dobutamine stress echocardiography shows failure to augment cardiac output at peak stress. There was no difference in cardiac output between baseline (panel A) and peak stress (panel B). The aortic valve was severely calcified (panel C). LVEF was severely reduced at peak stress (panel D: end systolic; panel E: end diastolic). LVEF, left ventricular ejection fraction

Echocardiography demonstrated severe LV systolic dysfunction, LVEF of 20-25%, and reduced stroke volume (Doppler LVOT [left ventricular outflow tract] stroke volume of 30 mL and stroke volume index of 19.6 mL/m^2^). The aortic valve was thickened and appeared calcified (Figure 1).

The peak aortic valve velocity was 2.05 m/sec and the mean gradient was 9 mmHg. The calculated AVA by the continuity equation was 0.8 cm^2^ (indexed valve area of 0.5 cm/m^2^). During infusion of low-dose dobutamine, the LVOT Doppler stroke volume increased to 33 mL and the calculated aortic valve area (AVA) did not change (Table 1).

**Table 1:** Measurements pre- and post-revascularization on dobutamine stress echocardiography LVOT VTI, left ventricular outflow tract velocity time integral; AV, aortic valve; AV VTI, aortic valve velocity time integral; AVA, aortic valve area

Variable	Pre-revascularization				Post-revascularization		
	Dobutamine dose (mcg/kg/min)				Dobutamine dose (mcg/kg/min)		
	Baseline	2.5	10	20	7.5	15	20
LVOT VTI (cm)	6.6	7.207	6.8	6.3	10.7	13.1	13.6
Stroke volume (mL)	30	33	31	31	48	59	62
AV peak pressure gradient (mmHg)	17.4	19.7	24	26.5	21	23	25
AV VTI (cm)	38.4	43.1	47.3	47.9	42.9	46.1	46.4
AVA (cm^2^)	0.8	0.7	0.7	0.6	1.1	1.3	1.3
Indexed AVA (cm^2^/m^2^)	0.5	0.5	0.5	0.4	0.70	0.8	0.8

**Figure 2:**
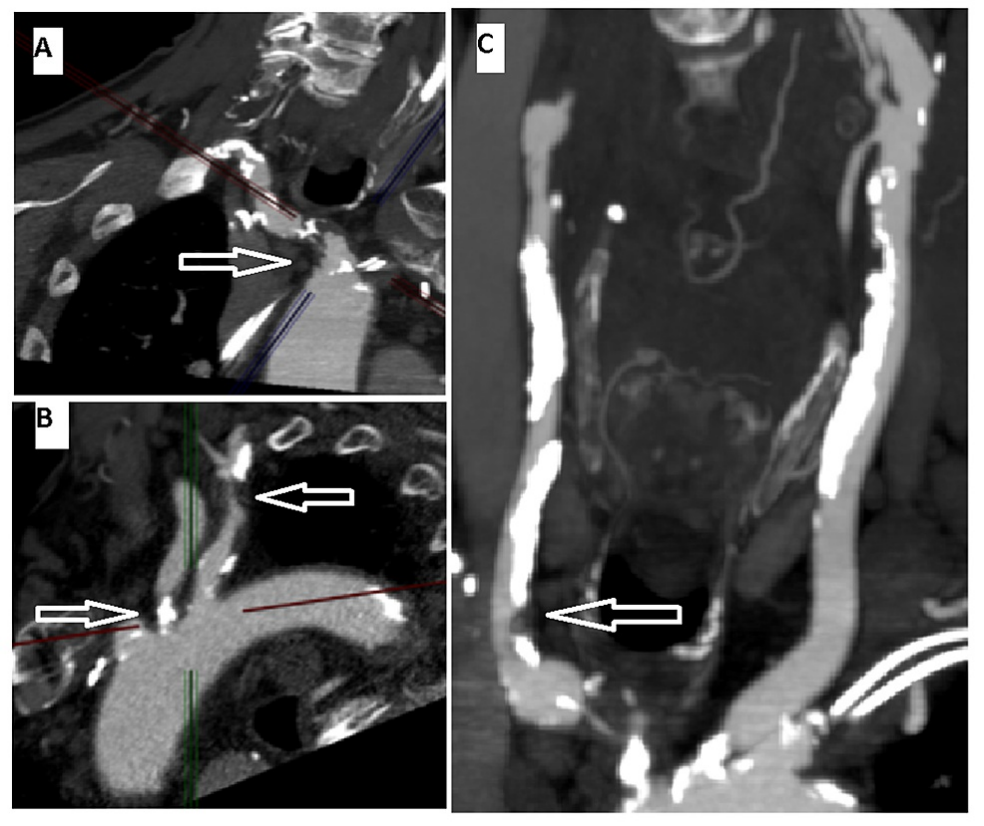
CTA of the head and neck reveals severe multivessel disease. CTA showed a severe proximal innominate artery stenosis (panel A, arrow), in addition to severe narrowing of the proximal left common carotid and subclavian arteries (panel B, arrows). In addition, there was moderate right common carotid artery stenosis (panel C, arrow). CTA, CT angiogram

The LVOT stroke volume then declined at higher doses, that is, a biphasic response suggestive of myocardial viability but not meeting standard criteria for flow reserve [2]. Two-dimensional images at peak stress demonstrated the areas of new distal septal, apical, apical lateral, and mid to distal inferior wall akinesis, concerning for LAD territory ischemia. CT angiogram of the head and neck showed extensive stenosis involving the right innominate artery, left common carotid artery, right common carotid artery, right internal carotid artery, and bilateral subclavian arteries extending to the axillary arteries (Figure 2).

**Figure 3:**
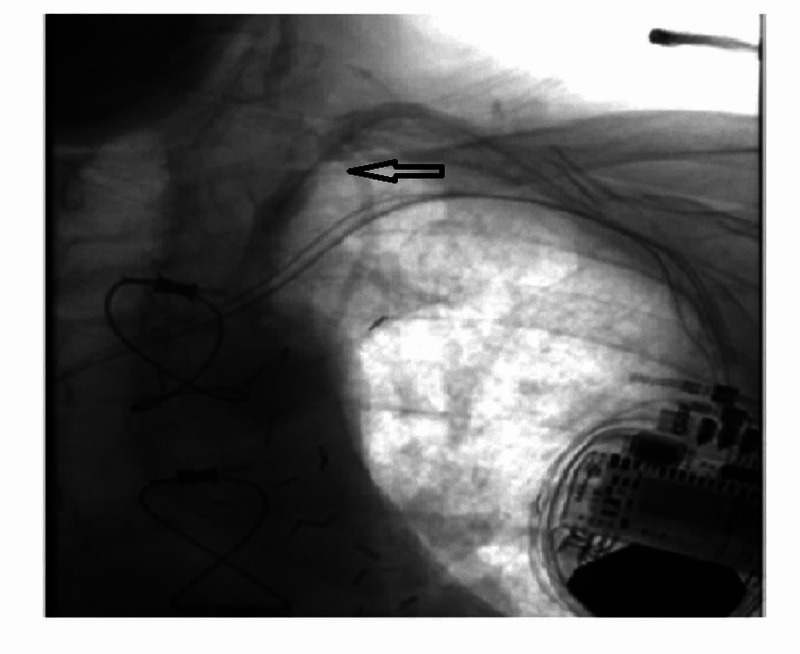
CT angiogram shows proximal left subclavian artery stenosis (arrow).

**Figure 4:**
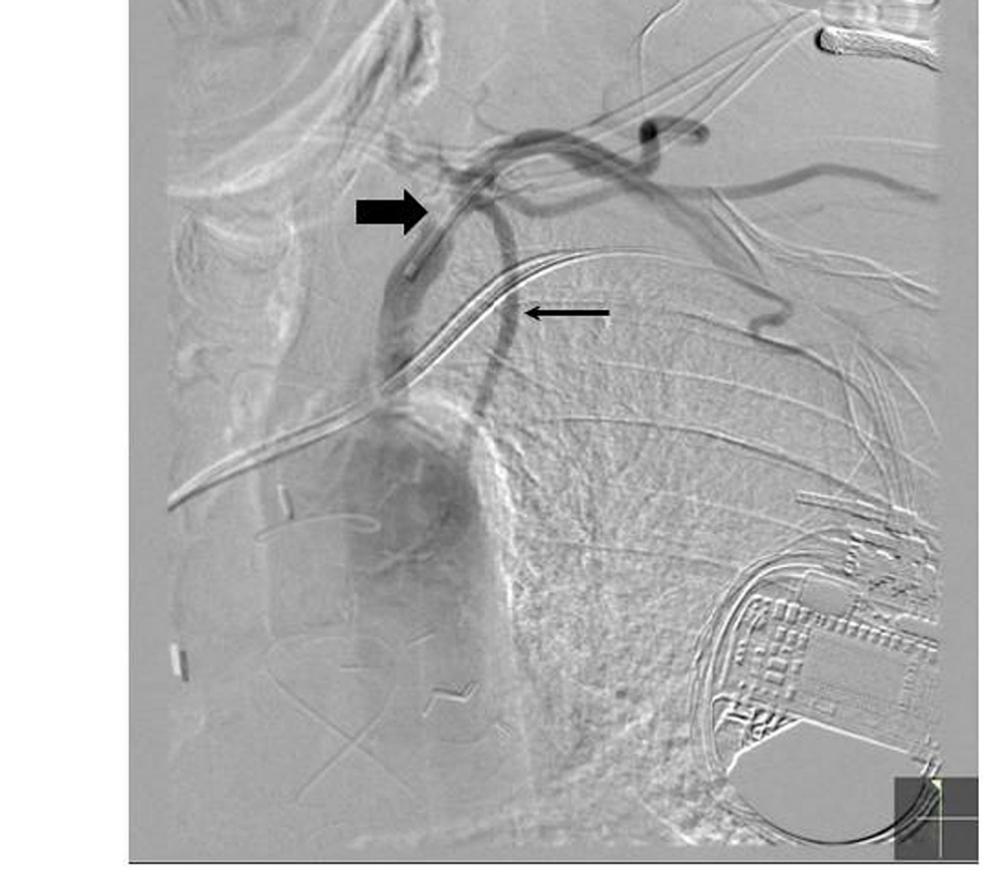
Cardiac catheterization shows severe left subclavian stenosis. Digital subtraction angiography image with catheter across severe left subclavian stenosis (thick arrow), just prior to the takeoff of the left internal mammary artery (thin arrow).

Coronary angiography revealed patent LIMA-LAD, SVG-OM1 (SVG to the first OM), and SVG-rPDA (SVG to the right PDA) grafts, but severe stenosis of the proximal left subclavian artery (70 mmHg gradient) proximal to the LIMA takeoff (Figures 3, 4).

Therefore, it was speculated that left subclavian arterial stenosis was a significant contributor to the LAD territory ischemia. A multidisciplinary team of cardiologists, interventional cardiologists, and vascular surgeons placed a 7-mm Dacron® bypass graft from the left common carotid artery to the left subclavian artery distal to the stenosis, reperfusing the LAD territory myocardium. This required intra-aortic balloon pump support and the patient remained on dobutamine post-operatively.

**Figure 5:**
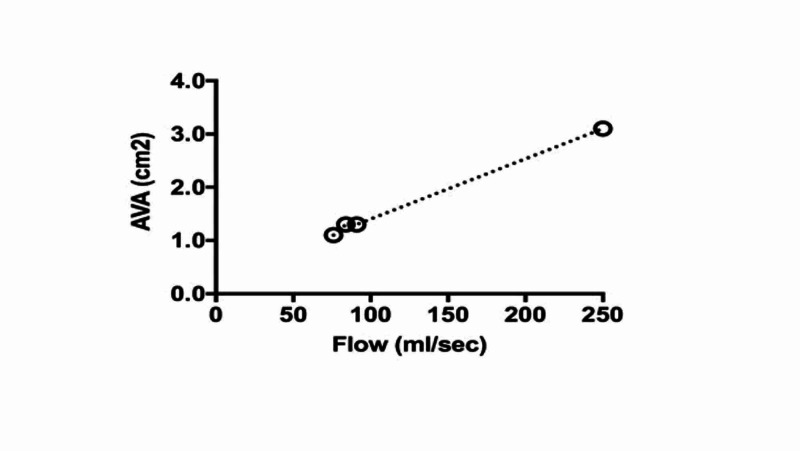
Projected effective orifice area at normal flow (250 mL/sec)

Repeat DSE five days following the bypass surgery demonstrated a higher LVOT stroke volume and AVA (48 mL and 1.1 cm^2^, respectively) on dobutamine 7.5 mcg/kg/min than present pre-operatively and a progressive, proportional rise in LVOT stroke volume and AVA (62 mL and 1.3 cm^2^, respectively) with increasing doses of dobutamine (up to 20 mcg/kg/min) (Table 1). This confirmed the diagnosis of AV pseudo stenosis. The projected valve area calculation showed an AVA of >3.0 cm^2^ at a flow of 250 mL/sec (Figure 5).

The patient was weaned off dobutamine and showed remarkable improvement in his symptoms. Patient was discharged to cardiac rehabilitation.

## Discussion

AS is the most common type of valvular heart disease in the developing world [3]. Severe AS is defined as a calculated AVA of ≤1 cm^2^, AVAi of ≤0.6 cm^2^/m^2^, mean pressure gradient (MPG) of ≥40 mmHg, and peak velocity of >4.0 m/s. A challenging subset of AS referred to as low-flow, low-gradient severe AS (LGSAS) can present with low calculated AVA, MPG, and peak velocity [4]. Based on Gorlin and Gorlin’s hydraulic equation, aortic valve EOA is directly proportional to flow and inversely proportional to velocity [5]. Hence, AVA can be artificially underestimated, leading to a diagnosis of severe AS in patients with only moderate AS [6].

Distinguishing TSAS and PSAS in the setting of LV dysfunction continues to be a challenging clinical situation. In TSAS, the reduced AVA causes a chronic elevation in afterload and may result in cardiomyopathy if the valve is not replaced. In contrast, patients with PSAS have only a mildly to moderately reduced AVA with a concomitant cardiomyopathy leading to a low-flow state and an erroneous diagnosis of severe AS [2]. As a result, patients with TSAS benefit clinically from transcatheter aortic valve implantation (TAVI) or aortic valve replacement (AVR), whereas patients with PSAS often find clinical improvement from addressing the cause of underlying cardiomyopathy [2]. Thus, correct distinction between these two entities is vital, as it will alter their clinical course. This case exemplifies how addressing the root cause for reduced flow reserve can allow physicians to definitively diagnose PSAS versus true stenosis [7]. In this rare case, the culprit was a stenotic subclavian vessel, which caused ischemia to the LAD territory through its effect on the LIMA-LAD graft. Revascularization allowed for return of flow reserve. A subsequent DSE confirmed the diagnosis of PSAS and allowed us to spare this patient from aortic valve replacement, which would have carried a high risk of mortality.

The ACC/AHA (American College of Cardiology/American Heart Association) updated guidelines on the management of valvular heart disease recommend using DSE to evaluate LGSAS (class 2a recommendation) [8].

DSE has been shown to be useful in distinguishing TSAS from PSAS in patients with good flow reserve; however, it fails to make this distinction in patients with no flow reserve, which represents 30-40% of patients with LGSAS [2,9]. Low-flow reserve is defined by <20% increase in stroke volume during DSE secondary to possible underlying afterload mismatch, coronary artery disease leading to a decrease in myocardial perfusion, or permanent myocardial damage from prior infarcts or fibrosis [2]. In the no-flow reserve state, identifying patients with TSAS and PSAS becomes an even more challenging task due to a lack of stroke volume augmentation. For these patients, other non-invasive modalities have been suggested, such as multi-slice CT scan [10], cardiac MRI (CMR) [11,12], PET scan [13], and B-type natriuretic peptide (BNP) levels [14].

In general, patients with true AS have static aortic valve leaflets and ample calcification. Cueff et al. suggested multi-slice CT scan as a reliable method for discerning TSAS from PSAS [10]. Their retrospective study found that an aortic valve calcium score of >1,650 arbitrary units (AU) measured using a multi-slice CT scan has 82% sensitivity and 80% specificity in differentiating TSAS from PSAS [10]. The absence of high calcium score is consistent with a functional aortic valve and suggests ventricular dysfunction as the primary cause of low transvalvular flow. The downside of cardiac CT scan is its inability to assess the flow velocity, and thus valve EOA cannot be estimated [12]. Garcia et al. studied the concordance of transthoracic echocardiography (TTE) and CMR in estimating EOA and found that CMR is a reliable method in estimating AS severity [12]. In a subanalysis of the TOPAS (Truly or Pseudo-Severe Aortic Stenosis) study, PET scans were used to study myocardial flow reserve (MFR) in patients with LGSAS and found that patients with TSAS have a lower MFR when compared to PSAS [13]. Furthermore, Bergler-Klein et al. found that BNP is significantly higher in patients with TSAS compared to those with PSAS, making BNP level an additional tool in evaluating these patients [14]. However, the utility of these approaches, while attaining moderate success in experimental settings, does not translate well to the clinical setting. These methods are cumbersome and expensive and, most importantly, often result in indeterminate results. Unfortunately, patients with pseudo AS have a significantly higher risk of mortality as compared with those with true AS in the surgical setting; however, similar outcomes have been demonstrated with TAVI [15].

**Figure 6:**
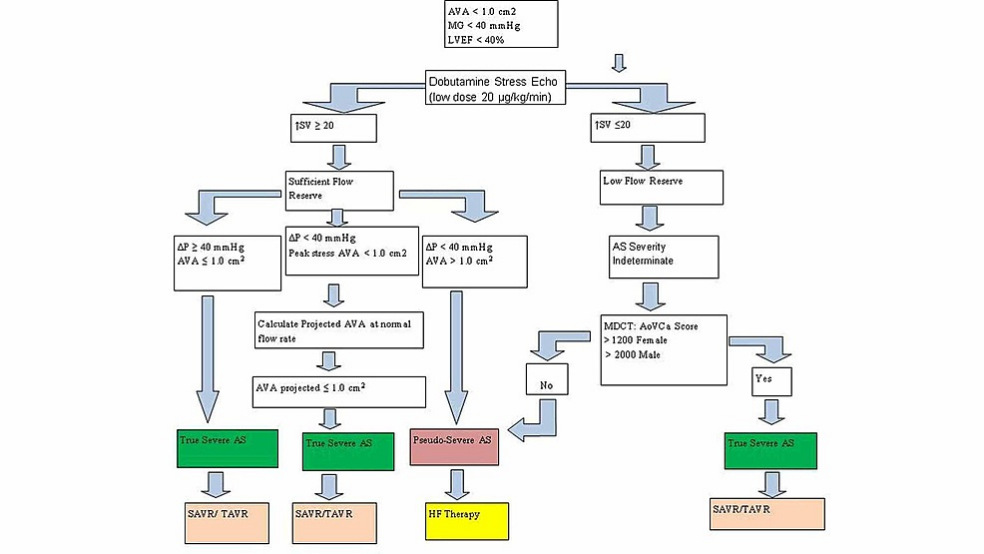
Algorithm for the management of low-flow, low-gradient aortic stenosis. Adapted from Dahou et al. [16] AS, aortic stenosis; AVA, aortic valve area; ∆P, change in pressure; HF, heart failure; LVEF, left ventricular ejection fraction; MDCT, multidetector CT scan; MG, mean gradient; SAVR, surgical aortic valve replacement; SV, stroke volume; TAVR, transcatheter aortic valve replacement

In patients with poor flow reserve, all potential causes of reversible LV dysfunction should be excluded. Coronary angiography is often indicated, as ischemic cardiomyopathy is the most common cause of poor contractile reserve. This includes careful assessment of venous and arterial conduits, in addition to exclusion of peripheral causes of reduced myocardial blood flow. In the case we presented, the contractile reserve was enhanced after restoration of blood flow to the LIMA graft site. A repeat DSE showed substantial increase in AVA and stroke volume, allowing us to definitively diagnose PSAS. An algorithm adapted from the ACC guideline can guide assessing the patient and provide appropriate treatment (Figure 6) [16].

## Conclusions

In patients with LF-LG AS, traditional criteria differentiating true stenosis versus pseudo stenosis using DSE is dependent on underlying flow reserve. Those without contractile reserve do not respond well to dobutamine stress, and the DSE is not useful. We present a unique case of a patient with a stenotic subclavian artery causing reversible ischemia to a LIMA graft. We restored blood flow to the LIMA by placing a graft from the internal carotid artery to a site distal to the stenotic subclavian. The contractile reserve was restored, and repeat DSE allowed us to definitively diagnose pseudo AS and spared the patient’s high-risk surgery.
